# Natural Carotenoids as Nanomaterial Precursors for Molecular Photovoltaics: A Computational DFT Study

**DOI:** 10.3390/molecules15074490

**Published:** 2010-06-24

**Authors:** Teresita Ruiz-Anchondo, Norma Flores-Holguín, Daniel Glossman-Mitnik

**Affiliations:** Departamento de Simulación Computacional y Modelado Molecular, Centro de Investigación en Materiales Avanzados, SC, Miguel de Cervantes 120, Complejo Industrial Chihuahua, Chihuahua, 31109, Mexico

**Keywords:** carotenoids, molecular structure, infrared spectrum, ultraviolet spectrum, dipole moment, polarizability, pKa, Conceptual DFT

## Abstract

In this work several natural carotenoids were studied as potential nanomaterial precursors for molecular photovoltaics. M05-2X/6-31+G(d,p) level of theory calculations were used to obtain their molecular structures, as well as to predict the infrared (IR) and ultraviolet (UV-Vis) spectra, the dipole moment and polarizability, the pKa, and the chemical reactivity parameters (electronegativity, hardness, electrophilicity and Fukui functions) that arise from Conceptual DFT. The calculated values were compared with the available experimental data for these molecules and discussed in terms of their usefulness in describing photovoltaic properties.

## 1. Introduction

Photovoltaic (PV) solar cells provide clean electrical energy because the solar energy is directly converted into electrical energy without emitting carbon dioxide. Solar energy is essentially unlimited, free of charge and distributed uniformly to all human beings. Crystalline silicon solar cells have been extensively studied and used for practical applications, however the expensive raw material cost and the high amounts of energy necessary for their manufacture have led to high cost and long energy payback times, which have prevented the wide spread of PV power generation [1]. This makes the development of new molecular materials and nanostructures using organic heterocycles highly desirable.

Photoelectrochemical solar cells have attracted a lot of attention because of their potential application for low-cost solar electricity generation [2]. Dye-Senstized Solar Cells (DSSC) are a kind of photoelectrochemical solar cells composed of a mesoporous oxide semiconductor layer and a dye senstizer attached to the surface. Ruthenium derivatives have proved to be the best dye sensitizers, providing great energy conversion efficiency [3,4,5], however, the limited availability of Ru metal could be an impediment for the industrial development of these cells. Therefore there is a great incentive to develop metal-free organic dyes as sensitizers for DSSC, because they would have lower cost, a high molar absorption coefficient, a relatively simple synthesis procedure, or they could be available from natural sources [6,7,8,9].

For thousands of years, dyes have been obtained from natural sources, such as plants and animals. In spite of the fact that synthetic dyes have replaced many natural ones for commercial use, natural dyes still hold a fascination and are used extensively by artisans around the world [10]. Most of these dye-sensitizers are carotenoids, cyanines, hemicyanines, coumarins, porphyrins, squaraines, phtalocyanins, perylenes, *etc*. Carotenoids are organic pigments naturally occurring in the chromoplasts of plants and some other photosynthetic organisms such as algae, and in some types of fungi and bacteria, where they have diverse and important functions and actions [11].

Theoretical investigations of the physical and chemical properties of dye sensitizers are very important in order to disclose the relationship between the structure, properties and performance, and to help in the design and synthesis of new dye sensitizers [12].

The objective of this work is to report the results of our calculations of the molecular structures and properties of several natural carotenoids (crocetin, bixin, norbixin, transbixin, and retinoic acid) using a recently developed density functional method [13]. The studied compounds have several desirable characteristics related to their use in molecular photovoltaics, specially as a photosensitizers in Dye-Sensitized Solar Cells (DSSC). Moreover, all of them have carboxylic acid groups in their structures, which enables their anchoring on the surface of the semiconductor film electrode, and the injection of electrons into the conduction band of the semiconductor [14]. An additional study is performed on a small ZnO cluster in an attempt to reproduce the properties of the ZnO surface that acts as an electron acceptor in a DSSC.

## 2. Theory and Computational Details

All computational studies were performed with the Gaussian 03W [15] series of programs with density functional methods as implemented in the computational package. The equilibrium geometries of the molecules were determined by means of the gradient technique. The force constants and vibrational frequencies were determined by computing analytical frequencies on the stationary points obtained after the optimization to check if there were true minima. The basis sets used in this work were 6-31+G(d) and LANL2DZ (for their explanation see refs. [16,17,18,19]).

For what concerns the calculation of the gas-phase properties, we have chosen the hybrid meta-GGA M05-2X functional [13], which consistently provides satisfactory results for several structural and thermodynamic properties. Solvation energies were computed by the Integral Equation Formalism- Polarizable Continuum Model (IEF-PCM) [20], including the UAKS model and water as a solvent.

The calculation of the ultraviolet (UV-Vis) spectra of the carotenoids has been performed by solving the time-dependent DFT (TD-DFT) equations according to the method implemented in Gaussian 03W [16,21,22,23]. The equations have been solved for 10 excited states.

The infrared (IR) and ultraviolet (UV-Vis) spectra were calculated and visualized using the Swizard program [24]. In all cases the displayed spectra show the calculated frequencies and absorption or emission wavelengths.

The molecular dipole moment is perhaps the simplest experimental measure of charge distribution in a molecule. The accuracy of the overall distribution of electrons in a molecule is hard to quantify, since it involves all the multipoles. The polarizability *α* contributes to the understanding of the response of the system when the external field is changed, while the number of electrons N is kept fixed. The polarizability is calculated as the average of the polarizability tensor <*α>* = (*α_xx_* + *α_yy_* + *α_zz_*)/3.

The pKa of hydrogen atoms attached to oxygen atoms is calculated using the MOPAC 2009 program [25]. In this program, the pKa is calculated using the O-H distance calculated using PM6 [26], and a charge calculated using a method specifically designed to reproduce the charge for pKa.

Within the conceptual framework of DFT [27], the chemical potential *µ*, which measures the escape tendency of an electron from equilibrium is defined as:

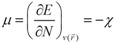
(1)
where 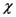 is the electronegativity. The global hardness *η* can be seen as the resistance to charge transfer:

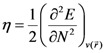
(2)


Using a finite difference approximation and Koopmans’ theorem [16,17,18,19], the above expressions can be written as:

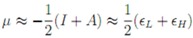
(3)

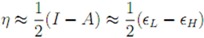
(4)
where 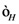 and 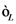 represent the energies of the highest occupied and the lowest unoccupied molecular orbitals (HOMO and LUMO), respectively.

The electrophilicity index ω represents the stabilization energy of the systems when it gets saturated by electrons coming from the surroundings:

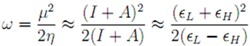
(5)


The validity of the Koopmans’ theorem within the DFT approximation is controversial. However, it has been shown [28] that although the KS orbitals may differ in shape and energy from the HF orbitals, the combination of them produces Conceptual DFT reactivity descriptors that correlate quite well with the reactivity descriptors obtained through Hartree-Fock calculations. Thus, it is worth to calculate the electronegativity, global hardness and global electrophilicity for the carotenoid molecules using both approximations in order to verify the quality of the procedures.

The condensed Fukui functions can also be employed to determine the reactivity of each atom in the molecule. The corresponding condensed functions are given by 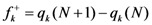 (for nucleophilic attack), 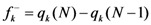 (for electrophilic attack), and 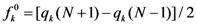 (for radical attack), where 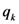 is the gross charge of atom 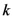 in the molecule. 

It is possible to evaluate condensed Fukui functions from single-points calculations directly, without resorting to additional calculations involving the systems with N-1 and N+1 electrons:

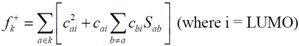
(6)
and:

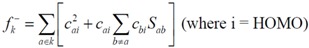
(7)
with *c_ai_* being the LCAO coefficients and *S_ab_* the overlap matrix. The condensed Fukui functions are normalized, thus 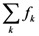 =1 and 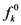 =[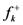 + 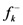]/2.

The condensed Fukui functions have been calculated using the AOMix molecular analysis program [29,30] starting from single-point energy calculations. We have presented, discussed and successfully applied the described procedure in our previous studies on different molecular systems [31,32,33].

The dual descriptor index has been defined [34,35] as:

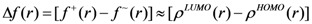
(8)


From the interpretation given to the Fukui function, one can note that the sign of the dual descriptor is very important to characterize the reactivity of a site within a molecule toward a nucleophilic or an electrophilic attack [34,35]. That is, if 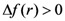, then the site is favored for a nucleophilic attack, whereas if 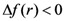 then the site may be favored for an electrophilic attack. Through a similar procedure, one finds that for the condensed dual descriptor [36]:

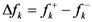
(9)


In the same line as before, the largest positive value of the condensed dual descriptor over an atom will indicate that this site will be the most prone to a nucleophilic attack, while the largest negative value will denote the atom most prone to an electrophilic attack. 

## 3. Results and Discussion

### 3.1. Crocetin

**Figure 1:**
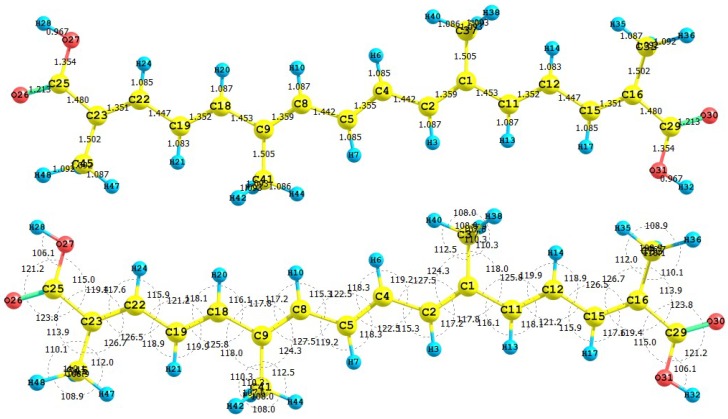
Interatomic bond distances (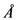) and bond angles (deg) for the crocetin molecule.

The carotenoid crocetin has been recently tested as a photosensitizer for DSSC [37]. Figure 1 presents the results for the equilibrium conformation of the neutral molecule of crocetin through a representation of the molecular structure of the molecule showing the interatomic bond lengths and bond angles calculated at the M05-2X/6-31+G(d,p) level of theory.

The infrared spectrum (IR) for the crocetin molecule calculated at the M05-2X/6-31+G(d,p) level of theory is presented by displaying the most intense calculated frequencies together with an explanation of the transition assignments. The calculated spectrum is in a qualitative good agreement with the experimental results. The most intense calculated frequencies are: 542.5 cm*^-^*^1^ = O27-H28 rocking, 776.8 cm*^-^*^1^ = O31-H32 rocking, 1002.6 cm*^-^*^1^ = C33-methyl waving, 1020.4 cm*^-^*^1^ = C7-H8, C5-H6, C3-H4 waving, 1044.2 cm*^-^*^1^ = C1-O27 stretching, 1143.5 cm*^-^*^1^ = C41-methyl waving, 1204.2, 1222.1 and 1235.8 cm*^--^*^1^ = C-H rocking, 1353.4 cm*^-^*^1^ = C1-O-27-H28 twisting, 1394.8 cm*^-^*^1^ = C26-C31-H32 twisting, 1416.9 cm*^-^*^1^ = C21-C19 stretching, 1683.6 cm*^-^*^1^ = C25-C23 stretching, 1708.8 cm*^--^*^1^ = C2-C3 stretching, 1732.9 cm*^-^*^1^ = C26-O30 stretching, 1827.3 cm*^--^*^1^ = C1-O29 stretching, 3228.4 cm*^-^*^1^ = O27-H28 stretching, and 3848.1 cm*^-^*^1^ = O31-H32 stretching.

The ultraviolet spectrum (UV-Vis) of the crocetin molecule calculated with the M05-2X/6-31+G(d,p) level of theory is presented by showing the principal transitions in Table 1. The wavelengths belonging to the HOMO-LUMO transition appear at 438.0 nm, while the experimental value from the spectrum taken in light petroleum is 450 nm, with a specific absorption coefficient of 4320 [38].

From the present calculations, the total energy, the total dipole moment and the isotropic polarizability of the ground state of crocetin at the M05-2X/6-31+G(d,p) level of theory are -1077.368 au, 0.0033 Debye and 333.13 Bohr^3^, respectively. The calculated pKa related to H28 and H32 is 4.544. These results could be of interest as an indication of the solubility and chemical reactivity of the studied molecule.

**Table 1:** Electronic transition states of crocetin (nm, eV, oscillator strengths (f), and transition assignments) as calculated with TD-DFT and the M05-2X/6-31+G(d,p) level of theory. Only the transition states with f > 0.02 are shown.

Number	Nm	eV	(f)	Assignment; H=HOMO, L=LUMO
1	438.0	2.83	3.2313	S H-0*→*L+0(+82%)
2	245.6	5.05	0.2678	S H-0*→*L+2(+32%) H-1*→*L+1(+25%)
				H-2*→*L+0(+17%)
3	241.9	5.13	0.0548	S H-2*→*L+0(+55%) H-0*→*L+2(+40%)
4	217.9	5.99	0.1235	S H-1*→*L+1(+59%) H-0*→*L+2(17%)
H-2 *→*L+0(15%)				

**Figure 2:**
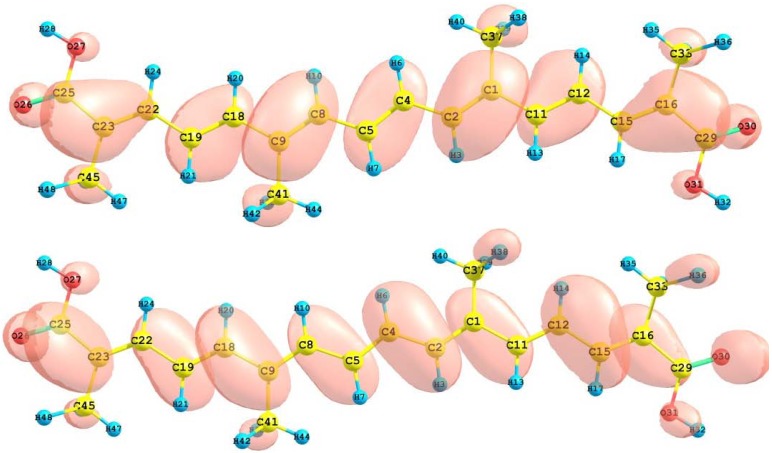
HOMO and LUMO of the crocetin molecule calculated with the M05-2X/6-31+G(d,p) level of theory.

The HOMO and LUMO of the crocetin molecule calculated at the M05-2X/6-31+G(d,p) level of theory are displayed in Figure 2. This can give us an idea of the reactivity of the molecule. The reactive sites can be identified through an analysis of the total and orbital densities. The representation of the calculated HOMO and LUMO densities in Figure 2 show that the electrophilic attack would occur preferentially at the C=C double bonds and the nucleophilic attack at the C-C single bonds.

The sites for electrophilic attack will be those atoms bearing a negative charge and where the Fukui function *f_k_^-^* is a maximum. These values and those coming from the dual descriptor index confirm that the sites for the electrophilic attack are the C15 and C22 atoms. The sites for potential nucleophilic attack would depend on the values of *f_k_*^+^ on the atoms with a positive charge density. The calculated results from the Fukui functions and the dual descriptor index show that the sites for nucleophilic attack will be the C1 and C9 atoms. 

The results for the vertical I and A of the crocetin molecule obtained through energy differences between the ionized and the neutral state, calculated at the geometry of the neutral molecule are I = 6.932 eV and A = 1.694 eV. The HOMO and LUMO energies are -6.589 eV and -2.075 eV, respectively. It can be seen that there is a good qualitative agreement between both results. The calculated values of the electronegativity, global hardness and global electrophilicity using the I and A are *𝜒* = 4.313 eV, *η* = 2.619 eV, and *ω* = 3.551 eV. Using the HOMO and LUMO energies, within the Koopmans’ theorem, the corresponding values are *𝜒* = 4.332 eV, *η* = 2.257 eV, and *ω* = 4.157 eV. Again, there is a good qualitative agreement for the reactivity parameters calculated through both procedures. It can be concluded that for the particular case of the crocetin molecule, the M05-2X/6-31+G(d,p) level of theory is able to predict the Conceptual DFT reactivity indices calculated through HOMO and LUMO energies as well as from the I and A obtained through energy differences with qualitative similar good accuracy.

### 3.2. Bixin, Norbixin and Transbixin

**Figure 3:**
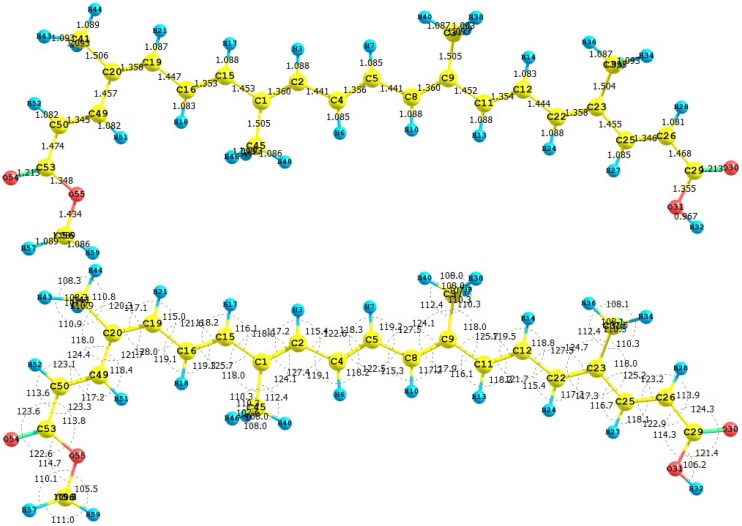
Interatomic bond distances (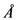) and bond angles (deg) for the bixin molecule.

**Figure 4:**
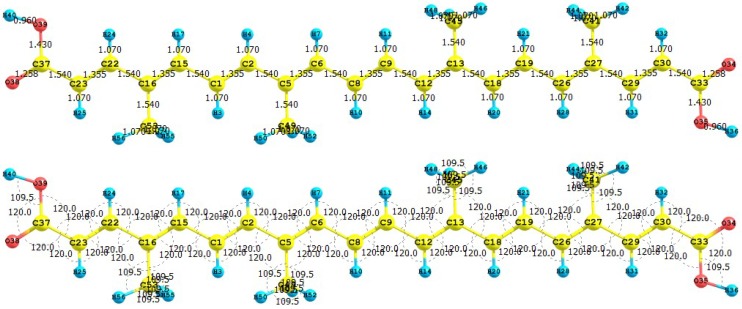
Interatomic bond distances (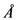) and bond angles (deg) for the norbixin molecule.

**Figure 5:**
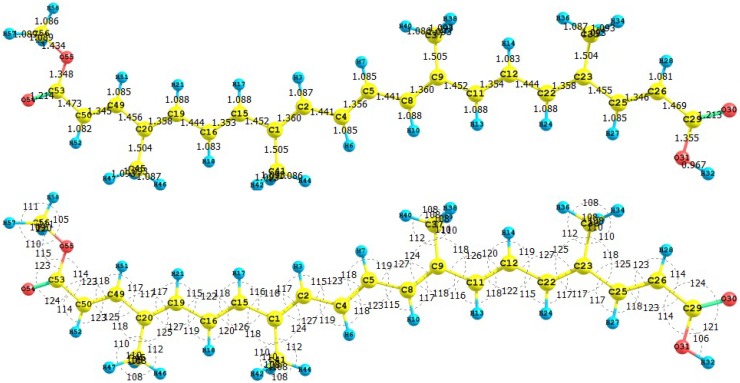
Interatomic bond distances (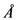) and bond angles (deg) for the transbixin molecule.

The apocarotenoids bixin (or *cis*-bixin), norbixin and transbixin have also been recently tested as photosensitizers for DSSC [39]. The results for the equilibrium conformations of the neutral molecules of bixin, norbixin and transbixin calculated at the M05-2X/6-31+G(d,p) level of theory are presented through a representation of the molecular structure of the molecule showing the interatomic bond lengths and bond angles in Figure 3, Figure 4 and Figure 5.

The infrared spectrum (IR) for the bixin molecule calculated with the M05-2X/6-31+G(d,p) level of theory is displayed by showing the most intense calculated frequencies and the corresponding assignments, which are: 611.0 cm^-1^ = O3-H4 rocking. 1040.4 cm^-1^ = methyl rocking, 1174.0 cm^-1^ = C1-O3-H4 waving, 1197.1 cm^-1^ = C-C stretching, 1214.1 cm^-1^ = C35-H36 rocking, 1330.2, 1370.7 and 1332.4 cm^-1^ = C-H rocking, 1462.2 cm^-1^ = C1-O3-H4 waving, 1662.8 cm^-1^ = C12-C14 and C26-C28 stretching, 1698.6 cm^-1^ = C30-C32 stretching, 1725.1 cm^-1^ = C5-C7 stretching, 1698.6 cm^-1^= C16-C17 and C23-C25 stretching, 1823.6 cm^-1^ = C33-C35 stretching, 1840.5 cm^-1^ = C1-O2 stretching, 3192.4 cm^-1^ = C-H stretching, 3228.0 cm^-1^ = methyl C-H asymmetric stretching, and 3622.2 cm^-1^ = O3-H4 stretching.

The most intense calculated frequencies in the infrared spectrum (IR) for the norbixin molecule calculated at the M05-2X/6-31+G(d,p) level of theory are: 556.9 cm^-1^ = O28-H29 rocking, 678.5 cm^-1^ = O49-H49 rocking, 1032.2 cm^-1^ = C-H rocking, 1034.6, 1078.6 and 1055.3 cm^-1^ = methyl waving, 1065.7 cm^-1^ = C47-O48 stretching, 1181.1 cm^-1^ = C1-O28 stretching, 1200.0 cm^-1^ = C-H waving, 1394.1 cm^-1^ = C1-C2 stretching, 1670.3 cm^-1^ = C20-C22 stretching, 1694.9 cm^-1^ = C23-C46 stretching, 1720.8 cm^-^^1^ = C11-C2 stretching, 1723.8 cm^-1^ = C25-C26 stretching, 1727.9 cm^-1^ = C2-C3 stretching, 1792.2 cm^-1^ = C47-O50 stretching, 1838.8 cm^-1^ = C1-O27 stretching, 3242.1 cm^-1^ = O28-H29 stretching, and 3855.3 cm^-1^ = O48-H49 stretching.

The most intense calculated frequencies in the infrared spectrum (IR) for the transbixin molecule calculated with the M05-2X/6-31+G(d,p) level of theory are: 650.7 cm^-1^ = O36-H37 rocking, 1014.3 cm^-1^ = C-H rocking, 1031.7 and 1035.0 cm^-1^ = methyl waving, 112.1 cm^-1^ = C1-O38 stretching, 1194.7 cm^-1^ = C33-H32 rocking, 1246.6, 1328.5, 1333.4 and 1343.6 cm^-1^ = C-H rocking, 1394.0 cm^-1^ = C1-O36-H37 scissoring, 1462.6 cm^-1^ = methyl waving, 1670.8 cm^-1^ = C9-C11 stretching, 1696.7 cm^‑1^ = C27-C29 stretching, 1721.5 cm^-1^ = C13-C14 stretching, 1724.6 cm^-1^ = C2-C4 stretching, 1822.5 cm^-1^ = C34-O38 stretching, 1838.6 cm^-1^ = C1-O35 stretching, and 3850.7 cm^-1^ = O36-H37 stretching.

The ultraviolet spectrum (UV-Vis) of the bixin, norbixin, and transbixin molecules calculated with the M05-2X/6-31+G(d,p) level of theory are presented by showing the principal transitions in Table 2, Table 3 and Table 4. The wavelength corresponding to the HOMO-LUMO transition will appear at 471.6 nm for bixin, 476.9 nm for norbixin, and 476.2 nm for transbixin. The reported experimental absorptions occur at 456 nm for bixin in light petroleum, and at 474.0 nm for norbixin in chloroform [35].

**Table 2:** Electronic transition states of bixin (nm, eV, oscillator strengths (f), and transition assignments as calculated with TD-DFT and the M05-2X/6-31+G(d,p) level of theory. Only the transition states with f > 0.02 are shown.

Number	nm	eV	(f)	Assignment; H=HOMO, L=LUMO
1	471.6	2.63	3.7551	S H-0*→L*+0(+81%) H-1*→L*+1(+5%)
2	346.1	3.58	0.1111	S H-0*→L*+1(+67%) H-1*→L*+0(+12%)
3	265.6	4.67	0.2404	S H-2*→L*+0(+59%) H-0*→L*+2(14%)
4	243.4	5.09	0.1220	S H-1*→L*+1(+52%) H-2*→L*+0(+26%)
				H-0*→L*+2(10%)
5	235.1	5.27	0.1639	S H-0*→L*+3(+40%) H-1*→L*+2(29%)
6	227.1	5.46	0.0405	S H-3*→L*+0(+45%) H-0*→L*+3(+21%)
				H-2*→L*+1(+18%)

**Table 3:** Electronic transition states of norbixin (nm, eV, oscillator strengths (f), and transition assign-ments as calculated with TD-DFT and the M05-2X/6-31+G(d,p) level of theory. Only the transition states with f > 0.02 are shown.

Number	nm	eV	(f)	Assignment; H=HOMO, L=LUMO
1	476.9	2.60	3.9337	S H-0*→L*+0(+82%) H-1*→L*+1(5%)
2	276.0	4.49	0.3509	S H-0*→L*+2(+46%) H-1*→L*+1(+25%)
3	265.6	4.67	0.1728	S H-2*→L*+0(+58%) H-0*→L*+2(+29%)
				H-1*→L*+1(5%)
4	243.3	4.63	0.1128	S H-1*→L*+1(+53%) H-2*→L*+0(+23%)
				H-0*→L*+2(14%)

**Table 4:** Electronic transition states of transbixin (nm, eV, oscillator strengths (f), and transition assignments as calculated with TD-DFT and the M05-2X/6-31+G(d,p) level of theory. Only the transition states with f > 0.02 are shown.

Number	nm	eV	(f)	Assignment; H=HOMO, L=LUMO
1	476.2	2.60	3.9436	S H-0*→L*+0(+82%) H-1*→L*+1(5%)
2	275.7	4.50	0.3689	S H-0*→L*+2(+45%) H-1*→L*+1(+25%)
				H-2*→L*+0(6%)
3	265.5	4.67	0.1626	S H-2*→L*+0(+59%) H-0*→L*+2(+29%)
4	243.0	5.10	0.1156	S H-1*→L*+1(+53%) H-2*→L*+0(+22%)
				H-0*→L*+2(14%)

The total energy, the total dipole moment, the isotropic polarizability of the ground state calculated at the M052-2X/6-31+G(d,p) level of theory and the pKa calculated with MOPAC 2009 and PM6 for the bixin, norbixin, and transbixin molecules are presented in Table 5.

**Table 5:** Total energy E, dipole moment *μ*, polarizability *α*, calculated with the M05-2X/6-31+G(d,p) level of theory, and pKa calculated with MOPAC 2009 and PM6.

Molecule	E (a.u.)	*μ* (Debye)	*α* (Bohr^3^)	pKa
Bixin	-1271.461	1.88	477.84	4.784
Norbixin	-1232.061	1.43	466.17	4.768
Transbixin	-1271.462	1.02	468.84	4.778

**Figure 6:**
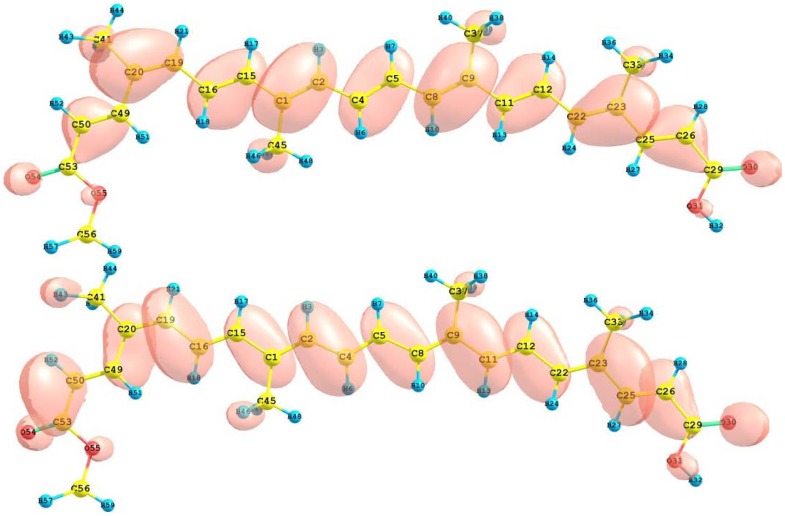
HOMO and LUMO of the bixin molecule calculated at the M05-2X/6-31+G(d,p) level of theory.

**Figure 7:**
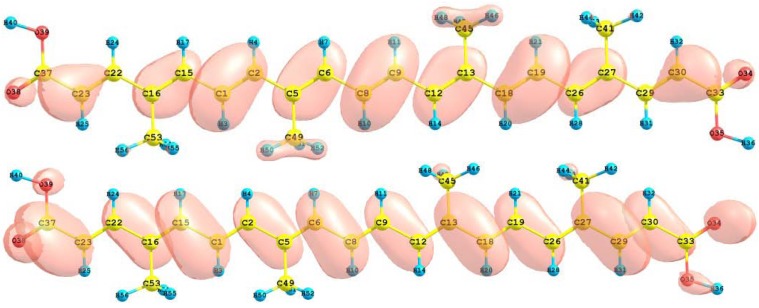
HOMO and LUMO of the norbixin molecule calculated at the M05-2X/6-31+G(d,p) level of theory.

**Figure 8:**
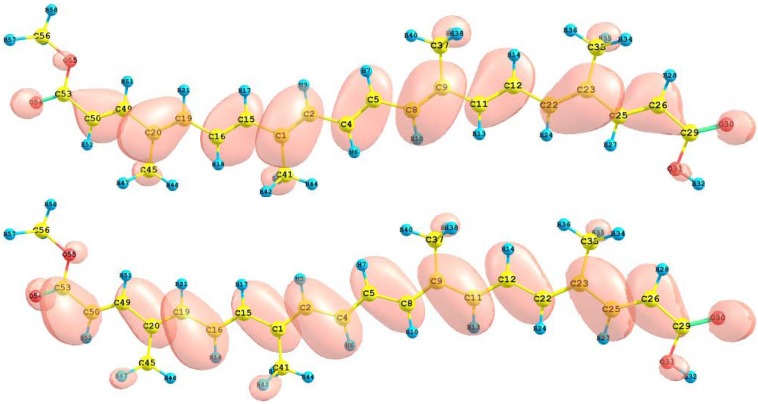
HOMO and LUMO of the transbixin molecule calculated with the M05-2X/6-31+G(d,p) level of theory.

The HOMO and LUMO orbital of the bixin, norbixin and transbixin molecules calculated with the M05-2X/6-31+G(d,p) level of theory are displayed in Figure 6, Figure 7 and Figure 8. The sites for electrophilic attack will be those atoms bearing a negative charge and where the Fukui function *f_k_^-^* is a maximum, while the sites for potential nucleophilic attack would depend on the values of *f_k_*^+^ on the atoms with a positive charge density. The calculated results are shown in Table 6.

**Table 6:** Electrophilic and nucleophilic attack sites for bixin, norbixin, and transbixin through Fukui functions and condensed dual descriptors calculated with the M05-2X/6-31+G(d,p) level of theory and the AOMix program. The value of the condensed dual descriptor (x 100) over the atom is shown in parentheses.

Molecule	Bixin	Norbixin	Transbixin
Electrophilic attack site	C11 (-2.72)	C23, C29 (-4.61)	C22 (-2.33)
Nucleophilic attack site	C9 (3.04)	C5, C13 (5.77)	C9 (2.83)

The results for the vertical IP and A of the bixin, norbixin and transbixin molecules obtained through energy differences between the ionized and the neutral state, calculated at the geometry of the neutral molecule, the HOMO and LUMO energies, and the calculated values of the electronegativity, global hardness and global electrofilicity using the I and A, and using the HOMO and LUMO energies, within the Koopmans’ theorem, are displayed in table 7. The agreement between the results of both groups of Conceptual DFT reactivity descriptors is qualitatively correct for the three molecules, being the for bixin very good. The ionization potentials I are better described using the Koopmans’ theorem approximation than the electron affinities A, and this can be ascribed as the main source of discrepancies.

**Table 7:** Ionization potential I, Electron affinity A, HOMO energy, LUMO energy, electronegativity, global hardness and global electrophilicity of bixin, norbixin and transbixin calculated with the M05-2X/6-31+G(d,p) level of theory and Eqns. 3, 4 and 5. The first group of Conceptual DFT reactivity descriptors corresponds to calculations based on energy differences, while the second group belongs to results based on HOMO and LUMO energies.

Molecule	Bixin	Norbixin	Transbixin
I (eV)	6.679	7.091	6.684
A (eV)	1.773	1.310	1.824
ℇ*_HOMO_* (eV)	-6.368	-6.727	-6.377
*ℇ_LUMO_* (eV)	-2.099	-1.718	-2.144
*𝜒* (eV)	4.226	4.201	4.254
*η*(eV)	2.453	2.891	2.430
*ω*(eV)	3.640	3.052	3.724
*𝜒* (eV)	4.234	4.223	4.261
*η* (eV)	2.135	2.505	2.117
*ω*(eV)	4.198	3.560	4.288

### 3.3. Retinoic acid

**Figure 9:**
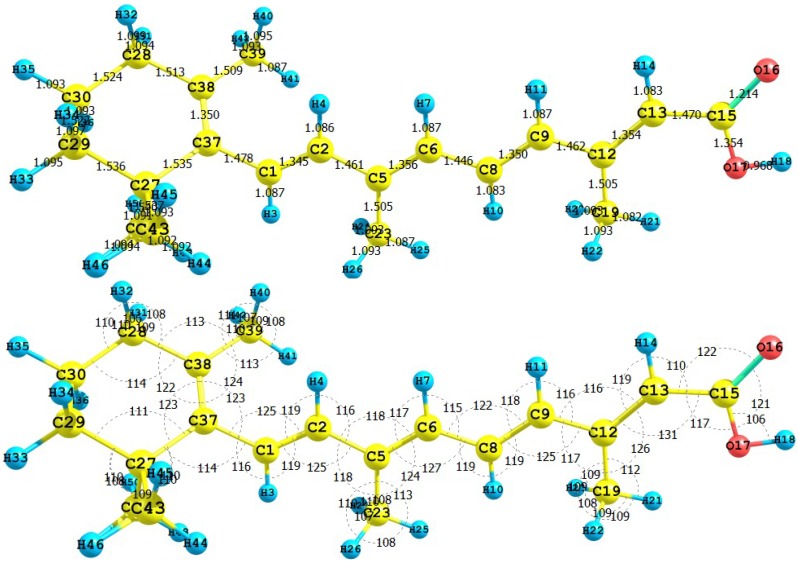
Interatomic bond distances (*
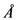
*) and bond angles (deg) for the retinoic acid molecule.

Retinol, also known as vitamin A, is essential for life. Retinol in the human body is produced by a series of metabolic processes that begin with dietary intake of *β*-carotene and retinyl esters. Retinoic acid (RA), the oxidized form of Vitamin A, is one of the most potent physiological metabolites of retinol. Retinoic acid has also been tried as a sensitizer for DSSC [40]. The results for the equilibrium conformation of the neutral molecule of retinoic acid calculated with the M05-2X/6-31+G(d,p) level of theory through a representation of the molecular structure of the molecule showing the interatomic bond lengths and bond angles are presented in Figure 9.

The infrared spectrum (IR) for the retinoic acid molecule calculated with the M05-2X/6-31+G(d,p) level of theory are presented by showing the most intense calculated frequencies and their assignments, which are: 609.3 cm^-1^ = O41-H42 rocking, 1021.9 cm^-1^ = C-H rocking, 1193.1 cm^-1^ = C39-O41 stretching, 1210.6 cm^-1 ^= C-C stretching, 1263.9 and 1404.8 cm^-1^ = C-H rocking, 1702.5 and 1725.3 cm^-1^ = C-C asymmetric stretching, 1824.0 cm^-1^ = C39-O40 stretching, and 3833.9 cm^-1^ = O41-H42 stretching.

The ultraviolet spectrum (UV-Vis) of the retinoic acid molecule calculated with the M05-2X/6-31+G(d,p) level of theory is presented by showing the principal transitions in Table 8. The wavelength belonging to the HOMO-LUMO transition will appear at 360.7 nm.

**Table 8:** Electronic transition states of retinoic acid (nm, eV, oscillator strengths (f), and transition assignments as calculated with TD-DFT and the M05-2X/6-31G(d,p) level of theory. Only the transition states with f > 0.02 are shown.

Number	nm	eV	(f)	Assignment; H=HOMO, L=LUMO
1	360.7	3.44	1.7642	S H-0*→L*+0(+83%)
2	264.3	4.69	0.1850	S H-1*→L*+0(+76%) H-0*→L*+1(+6%)
3	237.6	5.22	0.1527	S H-0*→L*+1(+80%) H-1*→L*+0(8%)
4	221.1	5.61	0.0325	S H-2*→L*+0(+73%)
5	202.5	6.12	0.0224	S H-0*→L*+3(+40%) H-3*→L*+0(11%)
6	198.0	6.26	0.1169	S H-1*→L*+1(+22%) H-0*→L*+7(17%)

From the present calculations, the total energy, the total dipole moment and the isotropic polarizability of the ground state of retinoic acid with the M052-2X/6-31+G(d,p) level of theory are ‑929.349 au, 4.790 Debye and 176.53 Bohr^3^, respectively. The calculated pKa related to the H18 is 6.690. These results could be of interest as an indication of the solubility and chemical reactivity of the studied molecule.

**Figure 10:**
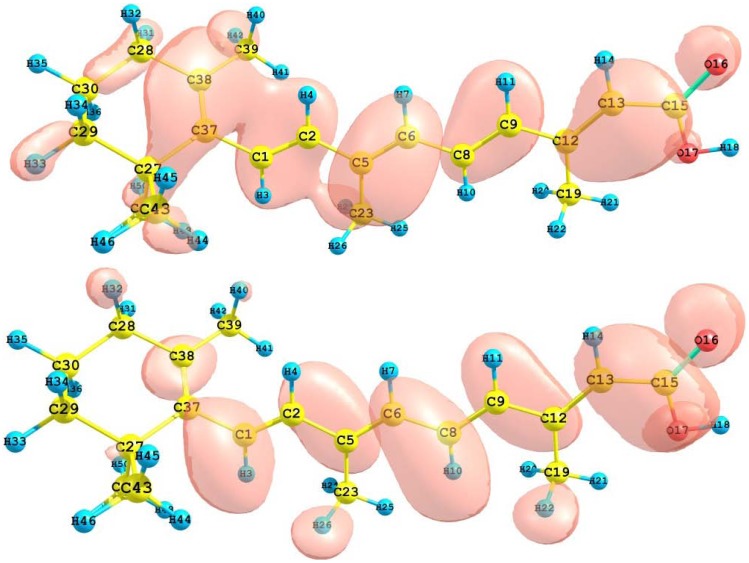
HOMO and LUMO of the retinoic acid molecule calculated with the M05-2X/6-31+G(d,p) level of theory.

The HOMO and LUMO of the retinoic acid molecule calculated with the M05-2X/6-31+G(d,p) level of theory are displayed in Figure 10. This can give us an idea of the reactivity of the molecule. The sites for electrophilic attack will be those atoms bearing a negative charge and where the Fukui function *f_k_^-^* is a maximum. These values as well as the results from the condensed dual descriptor confirm that the site for the electrophilic attack is the C12 atom. The site for potential nucleophilic attack would depend on the values of *f_k_*^+^ on the atoms with a positive charge density and where the condensed dual descriptor has the largest positive value. The calculated results show that the site for nucleophilic attack will be the C38 atom. 

The results for the vertical I and A of the retinoic acid molecule obtained through energy differences between the ionized and the neutral state, calculated at the geometry of the neutral molecule are I = 7.221 eV and A = 0.942 eV. The HOMO and LUMO energies are -7.986 eV and -1.445 eV, respectively. There is a good qualitative agreement between both results. The calculated values of the electronegativity, global hardness and global electrophilicity using the I and A are *𝜒* = 4.082 eV, *η* = 3.140 eV, and *ω* = 3.303 eV. Using the HOMO and LUMO energies, within the Koopmans’ theorem, the corresponding values are *𝜒* = 4.716 eV, *η* = 3.271 eV, and *ω* = 3.399 eV. Again, there is a good qualitative agreement for the reactivity parameters calculated through both procedures. It can be concluded that also for the particular case of the retinoic acid molecule, the M05-2X/6-31+G(d,p) level of theory is able to predict the Conceptual DFT reactivity indices calculated through HOMO and LUMO energies with the same degree of accuracy as the results from energy differences.

### 3.4. Zinc oxide (ZnO)

In its simplest configuration, the dye-sensitized solar cell (DSSC) is comprised of a transparent conducting glass electrode coated with porous nanocrystalline TiO_2_, dye molecules attached to the surface of the TiO_2_, an electrolyte containing a reduction-oxidation couple such as I*^-^*/ I*^-^*_3_ and a catalyst coated counter-electrode. Upon illumination, the cell produces an overvoltage and current through an external load connected to the electrodes. Due to the energy level positioning in the system, the cell is capable of producing voltage between its electrodes and across the external load. The maximum theoretical value for the photovoltage at an open circuit condition is determined by the potential difference between the conduction band edge of the TiO_2_ and the redox potential of the I*^-^*/I*^-^*_3_ pair in the electrolyte. The operation of the cell is regenerative in nature, since no chemical substances are neither consumed nor produced during the working cycle [41].

At the heart of the system is a mesoporous oxide layer composed of nanometer-sized particles which have been sintered together to allow for electronic conduction to take place. The material of choice has traditionally been TiO_2_ (anatase), although alternative wide band gap oxides such as ZnO, and Nb_2_O_5_ have also been investigated [42].

Zinc oxide (ZnO) has a large application potential owing to its diverse physical properties and the fine-tuning in the preparation process. Historically, ZnO has been used for a long time as pigment and protective coatings on metal surfaces. Its wide band gap of 3.2 eV at room temperature has rendered the use as protective UV-absorbing additive in everything from skin creams to advanced plastic and rubber composites. ZnO is an attractive material for nanoscale optoelectronic devices, as it is a wide-band gap semiconductor with good carrier mobility and can be doped both n and p-type. The electron mobility is much higher in ZnO than in TiO_2_, while the conduction band edge of both materials is located at approximately the same level [43].

**Figure 11:**
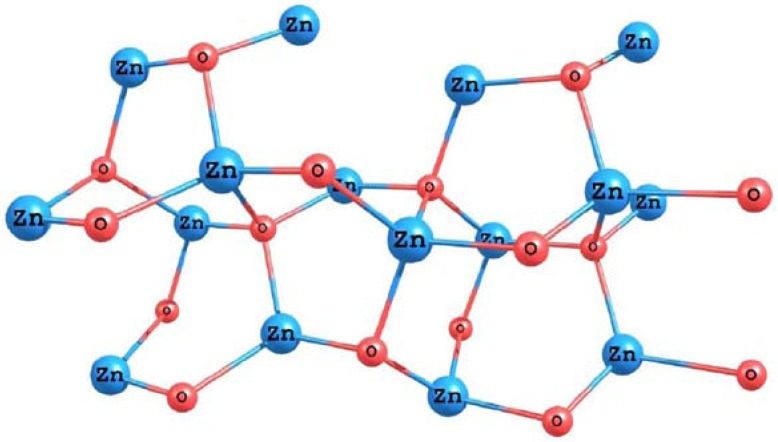
Molecular structure of the ZnO surface calculated with the M05-2X/LANL2DZ level of theory.

In order to simulate the ZnO surface, we have optimized the structure of a small cluster based on the (101) face of a zincite crystal. The results for the optimized structure of the system obtained using the M05-2X density functional and the LANL2DZ basis set are presented in Figure 11.

The results for the vertical I and A of the studied ZnO cluster obtained through energy differences between the ionized and the neutral state, calculated at the geometry of the neutral systems are I = 6.941 eV and A = 1.759 eV. The HOMO and LUMO energies are -6.286 eV and -2.340 eV, respectively. The calculated values of the electronegativity, global hardness and global electrophilicity using the I and A are *𝜒* = 4.350 eV, *η* = 2.591 eV, and *ω* = 3.652 eV. Using the HOMO and LUMO energies, within the Koopmans’ theorem, the corresponding values are *𝜒* = 4.313 eV, *η* = 1.973 eV, and *ω* = 4.714 eV. The calculated band gap is 3.95 eV. Indeed, this value is different from the experimental band gap not only due to the quality of the density functional employed, but also because we are considering a small cluster instead of the bulk system.

### 3.5. Photovoltaic properties

The conversion efficiency of the solar cell η is defined as the ratio of the generated maximum electric output power to the total power of the incident light P*_in_* [44]:

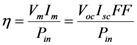
(8)
where I*_sc_* is the short-circuit current, V*_oc_* is the open-circuit voltage, and FF is the fill factor of the solar cell which is defined as:

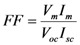
(9)


The photovoltaic parameters are evaluated under standard test conditions: the air mass (AM) 1.5 spectrum with an incident power density of 1,000 W/m^2^ and a temperature of 25 ^o^C. In order to improve the efficiency, it is necessary to maximize all the three photovoltaic parameters, such as V*_oc_*, I*_sc_* and FF [44].

The aim of device modeling is to develop a link between materials’ properties and the electrical device characteristics of a nanostructured solar cell. The goal of device modeling is to simulate the I-V curve of a nanostructured solar cell, both in dark and under illumination. From this, the main photovoltaic parameters of the solar cell are deduced [45]. However, this procedure is very involved both from a theoretical and computational point of view. Thus, we prefer to perform materials modeling, where materials parameters are studied and theoretically modeled based on physical and chemical phenomena and interactions.

For example, it is well known that there exists a relationship between the V*_oc_* and the interfacial band gap, which is defined as the difference between the HOMO of the donor (the absorbing dye) and the LUMO of the acceptor (the nanostructured metallic oxide). For a series of similar compounds, knowing the experimental efficiency, it should be possible to estimate the proportionality constant. However, the data for the carotenoids in our work have taken from different sources [37,39,40], with experiments performed in different conditions, and for this reason, it is not possible to do quantitative comparisons. On the contrary, some qualitative comparisons can be performed. For a given acceptor (ZnO in this case), the interfacial band gap will be larger when the HOMO of donor (the carotenoid) become larger. The results for the HOMO for the five carotenoids studied in this work are:

*retinoic acid > norbixin > crocetin > transbixin > bixin*

From this, it can be concluded that the retinoic acid caroteonid will be the more efficient for a DSSC based on nanostructured ZnO. A similar analysis, based on the calculated electronegativity *𝜒*, total hardness *η* and global elec-trophilicity *ω*, gives, respectively:

*retinoic acid > crocetin > transbixin > bixin > norbixin*

*retinoic acid > norbixin > crocetin > bixin > transbixin*

*transbixin > bixin > crocetin > norbixin > retinoic acid*

It can be concluded that if retinoic acid is the carotenoid which will render the best efficiency in a ZnO based DSSC according the results of the V*_oc_* related to the interfacial band gap, then the calculated values of electronegativity *𝜒*,total hardness *η* and global electrophilicity *ω* could also give an idea of the efficiency of the solar cell. That is, the larger the value of the Conceptual DFT reactivity parameters, the larger the efficiency of the DSSC. However, the opposite holds for the case of the global electrophilicity. Thus, it could be that the smaller the global electrophilicity, the larger the efficiency of the solar cell, and this deserves experimental confirmation.

Indeed, it must be recognized that a series of another factor could also be important, and that in our analysis we are not considering the variation of I*_sc_* with the calculated values. This suggests that a good theoretical relationship between the I*_sc_* and the electronic descriptors must be found. Moreover, the results of our work suggest that a Quantitative Structure-Property Relationship (QSPR) equation could be constructed in order to estimate the efficiency of the DSSC in terms of the HOMO, LUMO, and the conceptual DFT reactivity parameters.

## 4. Conclusions

In this work, the molecular structure and properties of five natural carotenoids that could be of interest in DSSC have been calculated using DFT through the M05-2X density functional and the 6-31+G(d,p) basis set.. Based on these structures, the IR spectra have been calculated, displayed, and the principal transitions have been explained. The UV-Vis of each molecule has been calculated with the same density functional and a 6-31+G(d,p) basis set. All calculations have been performed in the presence of water as a solvent. Every spectrum has been described by detailing ten excited states, and the HOMO-LUMO transition has been identified.

Some electronic properties like the total energy E, the dipole moment *µ* and the isotropic polarizability *𝛼* have also been calculated, with the pKa of the most acidic hydrogen in each has been determined through a procedure implement in MOPAC 2009. This could be of importance to understand the anchoring mechanism of the absorbing dye into the nanostructured ZnO.

A comparison between the ionization potential I and electron affinity A of the carotenoids calculated through two different procedures has been assessed in order to validate them. It could be concluded that, at least for the systems under study, the conceptual DFT reactivity parameters calculated directly from the HOMO and LUMO of the ground state of the carotenoids constitute a valid approach to values that can be used in estimating the efficiency of a solar cell.

The efficiency of the DSSC has been analyzed qualitatively in terms of the parameters which maximize the V*_oc_* related to the interfacial band gap, and the conclusions are: 1) the proposed model chemistry is capable of adequately describing the molecular structure and properties of the studied systems; 2) the retinoic acid is the best among the studied carotenoids; 3) it seems to be a rule that indicates that the larger the value of the of the global electronegativity and global hardness, and the smaller the global electrophilicity, then the larger efficiency of the solar cell. This deserves further computational studies and experimental confirmation, and work in this direction is being pursued in our laboratory. 
